# Integrative Faunistic, Ecological, and Molecular Study of *Harpirhynchus dusbabeki* and *Blankaartia acucutellaris* Associated with European Passerines

**DOI:** 10.3390/biology15171495

**Published:** 2026-09-02

**Authors:** Péter Takács, Tibor Hadarics, József Gyurácz, Péter Molnár, Enikő Horváth, Jenő Kontschán, Károly Erdélyi

**Affiliations:** 1 Disease Ecology and Wildlife Health Research Group, HUN-REN Veterinary Medical Research Institute, Hungária körút 21, 1143 Budapest, Hungary; 2 BirdLife Hungary, Határ út 72, 1038 Budapest, Hungary; 3 Berzsenyi Dániel Teacher Training Centre, Eötvös Loránd University, Károlyi Gáspár tér 4, 9700 Szombathely, Hungary; 4 Plant Protection Institute, HUN-REN Centre for Agricultural Research, P.O. Box 102, 1525 Budapest, Hungary; 5 Department of Plant Sciences, Albert Kázmér Faculty of Széchenyi István University, Vár tér 2, 9200 Mosonmagyaróvár, Hungary; 6 Department of Microbiology and Infectious Diseases, University of Veterinary Medicine, Hungária körút 23-25, 1143 Budapest, Hungary

**Keywords:** parasitic skin mites of birds, Bearded Reedling, *Harpirhynchus dusbabeki*, *Blankaartia acuscutellaris*

## Abstract

Parasitic skin mites associated with wild birds are understudied members of host–parasite systems in Europe despite their ecological importance, potential role as disease vectors, and effects on host fitness. Using a multidisciplinary approach combining faunistic, ecological, and molecular methods, this study investigated two mite species that parasitize passerine birds in Hungary, *Harpirhynchus dusbabeki* and *Blankaartia acuscutellaris*. Birds were examined during field sampling conducted between 2022 and 2024 at bird ringing stations and through a nationwide volunteer bird-ringing network. Parasites were identified morphologically, prevalence patterns were analyzed statistically, and mitochondrial genetic data were used to assess phylogenetic relationships. The occurrence of *H dusbabeki* was recorded in Hungary for the first time, and its host spectrum was found to extend to multiple host species beyond its previously known primary host, the Bearded Reedling. *Blankaartia acuscutellaris* infested several passerine species, with a significantly elevated prevalence in Bearded Reedlings and markedly higher prevalence among juveniles. Genetic analyses revealed close relatedness between the Hungarian and Slovakian populations of *B. acuscutellaris*, but showed distinct genetic separation from morphologically identified conspecifics from Laos, Asia. These findings improve the understanding of mite–passerine associations in Central–Eastern Europe and highlight the value of integrating ecological and molecular approaches to reveal the patterns of host exploitation and geographic differentiation in parasitic mites.

## Simple Summary

Parasitic mites that feed on the skin of wild birds are widespread, but their ecology remains poorly understood. These parasites may compromise the health, survival, and population dynamics of birds, yet information about their host range and distribution is scarce. In this study, we examined two mite species (*Harpirhynchus dusbabeki* and *Blankaartia acuscutellaris*) associated with songbirds in Hungary to understand which species they infest, how frequently infections occur, and whether geographically separated populations differ genetically. We found that *H. dusbabeki*, previously considered highly host-specific, was present on several additional bird species, indicating a broader host range than previously recognized. *Blankaartia acuscutellaris* was detected across multiple bird species but was most frequent in Bearded Reedlings, particularly in young individuals, suggesting that age-related behavior may influence the likelihood of infestation. Genetic analyses revealed clear differences between European and Asian populations of this mite species despite their isomorphic appearance, indicating previously unrecognized biological diversity. Our findings provide new insights into bird–parasite associations and highlight the contribution of combining field observations with modern biological methods to the understanding of wildlife health and ecological interactions.

## 1. Introduction

The role of ectoparasites in the general physiology and population dynamics of birds is non-negligible, given their potentially detrimental impact on survival and fitness [1]. These effects of parasitic arthropods—ticks, lice, fleas, and mites—are investigated to varying degrees. Parasitic mites represent a highly diverse yet underexplored group, especially in wild bird communities. Despite their ecological importance, knowledge on the distribution, host range, and epidemiological patterns of many taxa remains scarce, particularly in Central and Eastern Europe [2].

**Figure 1:**
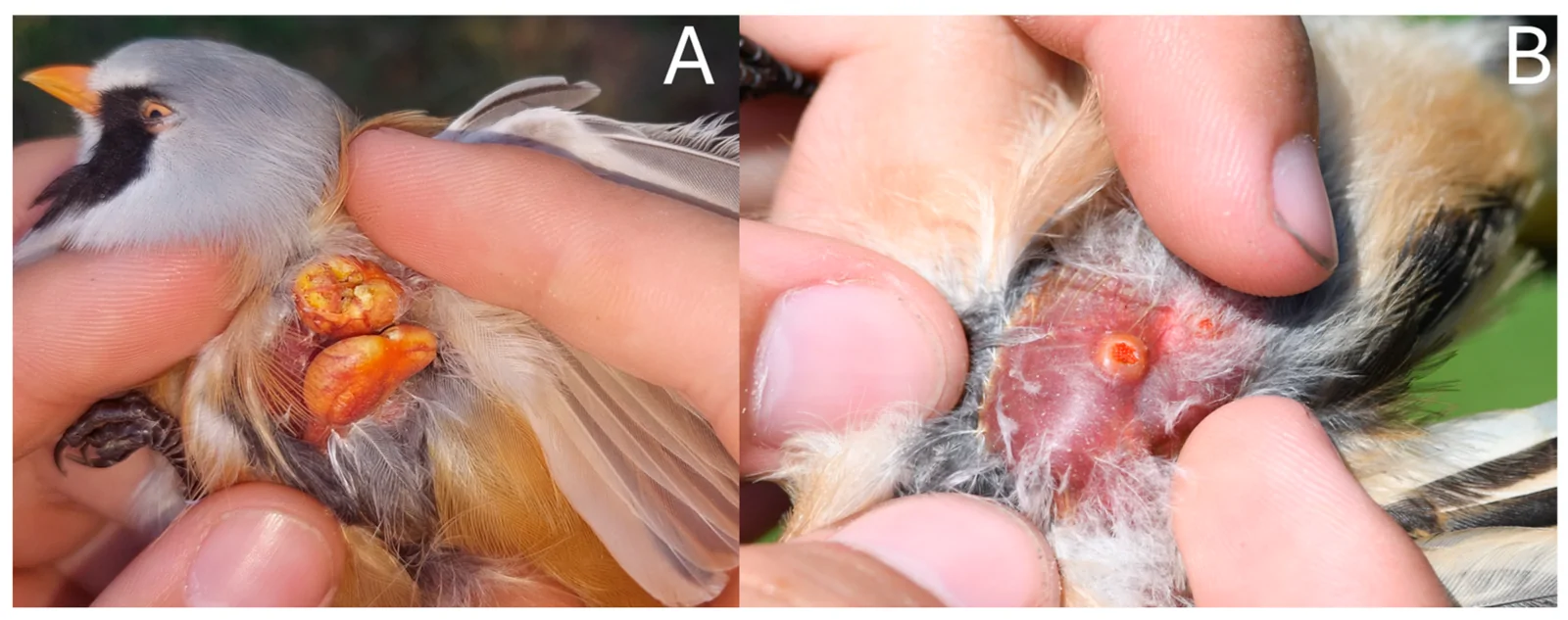
Skin lesions associated with *Harpirhynchus dusbabeki* (**A**) and *Blankaartia acuscutellaris* (**B**) infestation.

Mites associated with birds exhibit a wide range of ecological strategies [1]. For example, members of the family Harpirhynchidae Dubinin, 1957 (Arthropoda, Arachnida, Trombidiformes, Cheyletoidea, Harpirhynchidae) are permanent ectoparasites that inhabit their hosts’ skin and often exhibit high host specificity [3,4]. All life stages are obligate parasites and may induce extensive, yellowish nodular skin lesions, typically in the axillary, pectoral, and cloacal regions of birds (Figure 1), wherein mites feed and reproduce [5]. In Europe, two species of the genus have been reported, *Harpirhynchus nidulans* Nitzsch, 1818, and *H. dusbabeki* Bochkov & Literák, 2006 [3,6], and both are primarily associated with passerine hosts, especially Bearded Reedlings (*Panurus biarmicus*) [2,7]. However, their distribution and host range have been scarcely investigated, with the majority of records deriving from opportunistic observations of captured wild birds [3,5]. Moreover, key aspects of their biology, including transmission pathways, seasonal population dynamics, and their host spectrum, remain largely unknown.

Another understudied mite species is *Blankaartia acuscutellaris* Walch, 1922 (Arthropoda, Arachnida, Trombidiformes, Trombiculoidea, Trombiculidae). In contrast to harpirhynchid species, only the larval stage (called chiggers) is parasitic, while nymphs and adults are free-living in the soil or on low vegetation [8]. Larvae attach to the hosts in dense aggregations, forming so-called ‘mite islands’ on their skin (Figure 1), where they feed on tissue fluid and necrotized epithelium [9,10,11]. Although the Bearded Reedling is also considered a primary avian host, *B. acuscutellaris* has a broader host spectrum, including other passerines, reptiles, and rodents [12,13,14,15]. In addition, humans may serve as accidental hosts, since chiggers are the causative agents of trombiculiasis (trombiculosis), an acute dermatitis triggered by an allergic reaction to the mites’ salivary components [16]. While the disease is mainly reported from Asia, sporadic cases have also been documented in Europe, including Hungary [17]. Despite its potential negative impact on various vertebrate groups, the distribution and avian host spectrum of *B. acuscutellaris* in Central–Eastern Europe remain poorly documented.

Previous studies have primarily focused on a single host species or specific host–parasite systems, often providing only descriptive prevalence data. For instance, Henry et al. (2004) investigated *H. nidulans* exclusively in Bearded Reedlings [2], and, more recently, Trnka et al. (2023) reported *B. acuscutellaris* mite infestation in the same host species [18]. However, broader patterns of host specificity and parasite diversity across multiple host species remained poorly understood.

In addition, genomic data for these mite groups remain scarce, limiting our understanding of their phylogenetic relationships and population genetics. Integrating records of occurrence with ecological and molecular approaches is therefore essential to obtain a more comprehensive picture of host–parasite interactions [6].

In this study, we investigate two mite species that parasitize passerine birds in Central–Eastern Europe using a multidisciplinary approach that includes faunistic, ecological, and molecular methods. Specifically, we report new country and host records, characterize the host spectrum of the investigated mite species, analyze prevalence patterns with respect to host traits, and provide molecular data to explore the phylogenetic relationships of *B. acuscutellaris*. By integrating these aspects, our study aims to contribute to a more comprehensive understanding of lesser-known mite–passerine associations in Hungary and Central–Eastern Europe.

## 2. Materials and Methods

### 2.1. Study Area and Sampling

We collected field data on parasite occurrence in avian populations and parasite samples at a Constant Effort Site (CES) at Mekszikópuszta, a strictly protected area of the Fertő-Hanság National Park in Hungary, near the Austrian border. At the same time, volunteer bird ringers gathered parasites and parasite occurrence data from birds across Hungary. Bird capture, ringing, and biometric data collection were carried out in accordance with the standardized CES protocol [19]. Fieldwork was conducted between 2022 and 2024, from April to October, with two consecutive sampling days per month. During each sampling occasion, six mist nets (16 × 16 mm mesh size, 12 m long, 3.5 m high, 5 pockets) were used to capture birds.

After removal from the nets, birds were individually placed in cloth bags and transported to the ringing station. Species, age, and sex (when possible based on plumage characteristics) were determined based on Swensson (1992) [20]. Age categories were classified according to standard bird-ringing criteria for passerines as juvenile (first-calendar-year birds, hatched in the current year), full-grown (birds that had completed juvenile development but exact age could not be determined by plumage), or adult (individuals that have hatched in previous years). Each individual was banded with a uniquely coded metal ring on the left leg (tarsometatarsus).

Ectoparasite screening was performed by visually inspecting the skin after gently parting the feathers on the abdomen, breast, back, axillary regions, underwing area, and around the cloaca. Infection status (infected vs. non-infected) was recorded for each individual. In infected birds, characteristic lesions (e.g., orange “mite islands” or cyst-like structures) were documented, and parasite specimens were collected with fine needles and entomological forceps and then preserved in RNAlater solution (Thermo Fisher Scientific, Waltham, MA, USA) for subsequent molecular analyses.

To assess the host spectrum of the mite species studied, additional data were collected nationwide through a network of volunteer bird ringers coordinated by the Bird Ringing and Migration Study Group of BirdLife Hungary. Participating ringers were provided with a field protocol containing pictures of typical skin lesions for unambiguous identification. They recorded infection status, photographed lesions, and, when possible, collected voucher specimens of parasites following prior coordination. Ringing data were recorded and later retrieved from the Tringa Hungarian bird ringing database for analysis. Records accompanied by photographic evidence were treated as anecdotal but validated, whereas records with collected specimens were considered fully confirmed.

### 2.2. Parasite Identification

Mite specimens collected both at Mekszikópuszta and from volunteer bird ringers were identified to species level by Jenő Konstchán (Plant Protection Institute, HUN-REN Centre for Agricultural Research) based on external morphological characteristics. Specimens were slide-mounted on concave microscope slides and cleared in 85% lactic acid for five days prior to examination according to a routinely applied sample preparation procedure for entomological studies. After clearing, the mites were examined under a digital microscope (Leica DM 1000, Wetzlar, Germany).

Identification of Trombiculidae mites followed the taxonomic keys of Vercammen-Grandjean [21]. Diagnostic characters included the leg setation (arrangement, number, and structure of setae) and the morphology of the dorsal scutum, particularly its shape, structure, and setation.

Identification of *Harpirhynchus* species was based on the descriptions provided by Bochkov & Literák [6]. Key diagnostic features included the presence, absence, and number of specific setae. Particular attention was given to distinguishing *Harpirhynchus nidulans* from *H. dusbabeki*.

### 2.3. Molecular Assays and Phylogenetic Analysis

Total nucleic acid was extracted from three pooled *Blankaartia acuscutellaris* samples (50 individuals per pool) originating from three distinct, infected hosts using the Zymo Quick-DNA/RNA Viral Kit (Zymo Research, Irvine, CA, USA), following the manufacturer’s protocol. These samples were frozen at −80 °C until further processing.

A ~1200-base-pair-long fragment of the mitochondrial cytochrome c oxidase subunit I (COX1 or COI) gene and a ~1800 bp long fragment of the 18S ribosomal RNA were amplified using a nested PCR approach based on Klimov et al. (2018), with slight in-house modifications [22]. Detailed primer sequences and PCR conditions are provided in Supplementary Material File S1.

PCR products were visualized by 1.5% agarose gel electrophoresis, excised, and sequenced in both directions using the internal primers by Sanger sequencing. The obtained sequences were compared with reference sequences available in GenBank using BLAST 2.17.0 searches, focusing on entries annotated as *Blankaartia acuscutellaris* COX1 sequences [18].

Sequence editing and alignment with Clustal Omega were performed in the Geneious Prime 2023.1.2 software. Best-fitting models were identified using the Maximum Likelihood model selection tool in the MEGA 12.1.1 software package. Phylogenetic analysis of the 582bp long COX1 sequence alignment was performed using Bayesian inference implemented in the MrBayes plugin within the Geneious software package, utilising the GTR+G+I nucleotide substitution model [23]. American house dust mite (*Dermatophagoides farinae*) sequences (HQ823622 and GQ465336) were used as an outgroup, and a selection of sequences from closely related Trombiculid genera [24] obtained from GenBank provided further context for the analysis. To complement the phylogenetic analysis, pairwise nucleotide distances of the above COX1 sequence alignment were calculated in MEGA 12.1.1 using the Tamura–Nei substitution model. The Spearman Correlations of the resulting pairwise distance values were calculated and visualized as a heatmap using the online platform Heatmapper2 [25].

### 2.4. Statistical Analysis and Data Visualization

Host species- and age-related patterns of *Blankaartia acuscutellaris* infestation were analyzed using R version 4.5.1 (13 June 2025; R Core Team, 2025) within RStudio version 2025.05.1. The following packages were used for analyses and data visualization: *readxl*, *dplyr*, *ggplot2*, *ggtext*, *openxlsx*, with geographic visualization (Figure 3) generated using *giscoR*, *rnaturalearth*, and *ggplot2*. Species-specific prevalence of *B. acuscutellaris* infestation was calculated as the proportion of infested individuals within each species. Differences in prevalence between Bearded Reedlings and other host species, as well as between age classes (first-year vs. adult individuals) of Bearded Reedlings, were evaluated using Pearson’s chi-squared tests. Before analysis, expected cell frequencies were examined to verify that the assumptions of the chi-squared test were satisfied. Drawings of bird species depicted in Figure 5 and the Graphical Abstract were designed using OpenAI ChatGPT (GPT-5.5).

## 3. Results

### 3.1. Morphological Identification, Host Spectrum, and Prevalence of Mites

Over the study period, a total of 1919 birds belonging to 31 species were ringed and examined for ectoparasitic mites. The most frequently captured species was the Bearded Reedling (*n* = 820), followed by the Eurasian Reed Warbler (*Acrocephalus scirpaceus*; *n* = 427) and the Sedge Warbler (*Acrocephalus schoenobaenus*; *n* = 281). A complete summary of host species, sample sizes, and mite infestation records is provided in Table 1.

**Figure 2:**
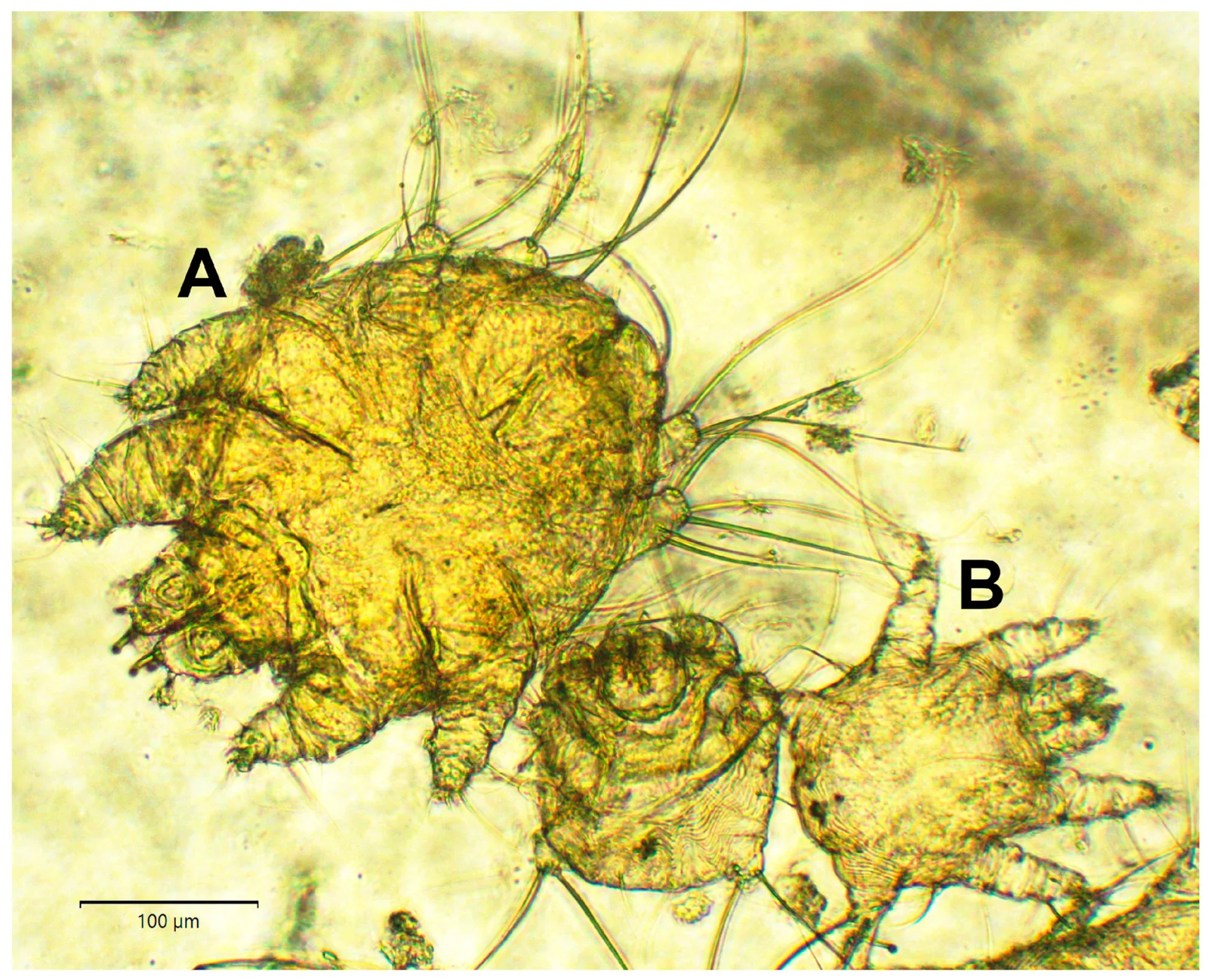
Adult (A) and larval specimens (B) of *Harpirhynchus dusbabeki* collected from a Bearded Reedling (Mekszikópuszta, Hungary, 2022).

From all captured birds exhibiting characteristic cyst-like skin lesions, we collected and identified *Harpirhynchus dusbabeki* adults and larvae, representing the first record of this species in Hungary (Figure 2).

**Figure 3:**
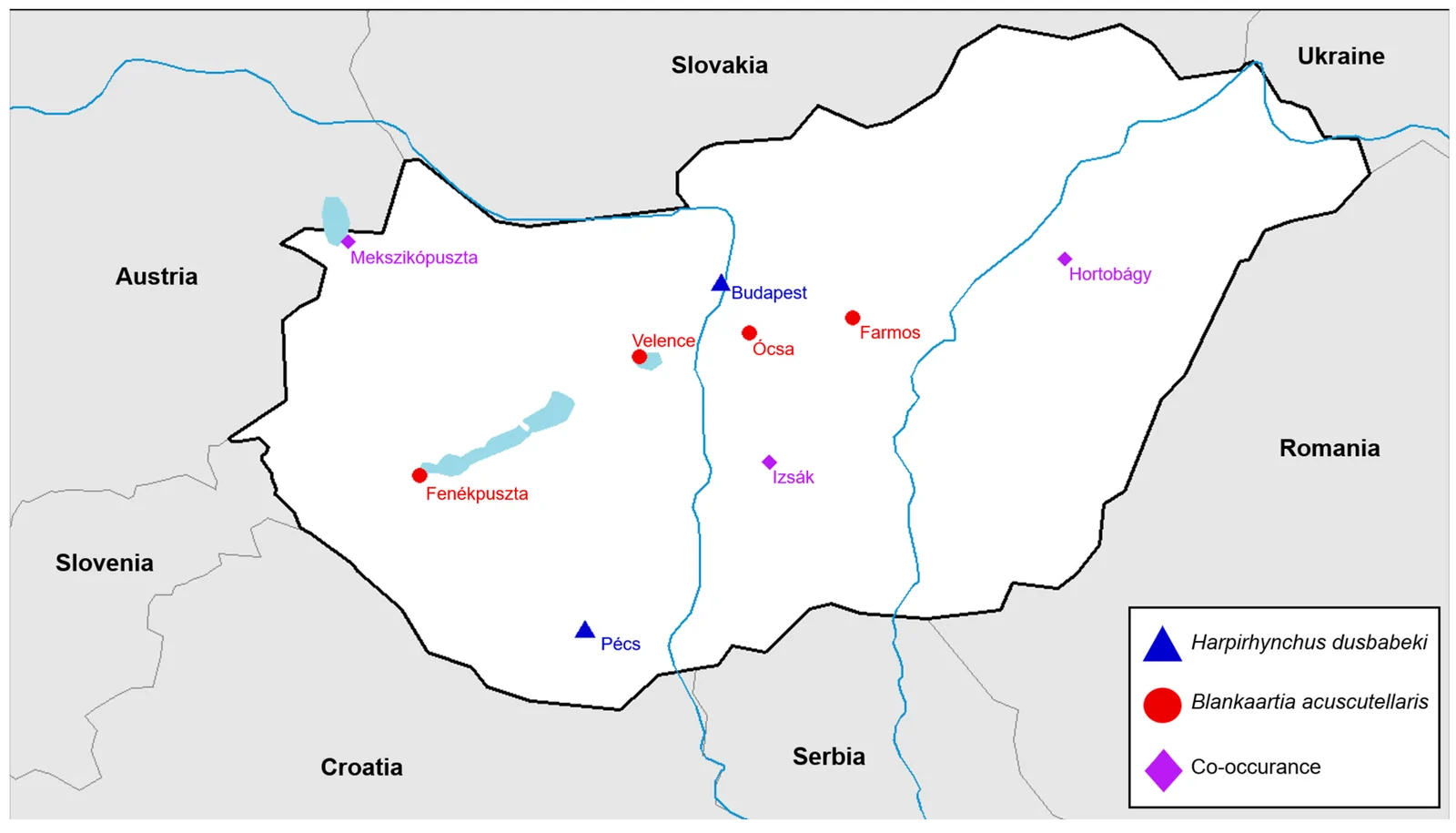
Occurrence of *H. dusbabeki* and *B. acuscutellaris* in Hungary, 2022–2024.

Occurrences of *H. dusbabeki* on Bearded Reedlings were recorded sporadically nationwide in Hungary. In Mekszikópuszta, only ten individuals were infested by this mite species throughout the whole study period. Based on nationwide bird-ringing data, we identified additional susceptible host species, thereby expanding our knowledge of *H. dusbabeki* host range. New host records include the Moustached Warbler (*Acrocephalus melanopogon*), recorded at Izsák; the Great Tit (*Parus major*), recorded in Budapest; and the Eurasian Siskin (*Spinus spinus*), recorded in Pécs (Figure 3). All infested birds were fully grown individuals. Based on these observations, the parasite occurs sporadically in the host populations and the studied habitats throughout Hungary.

**Figure 4:**
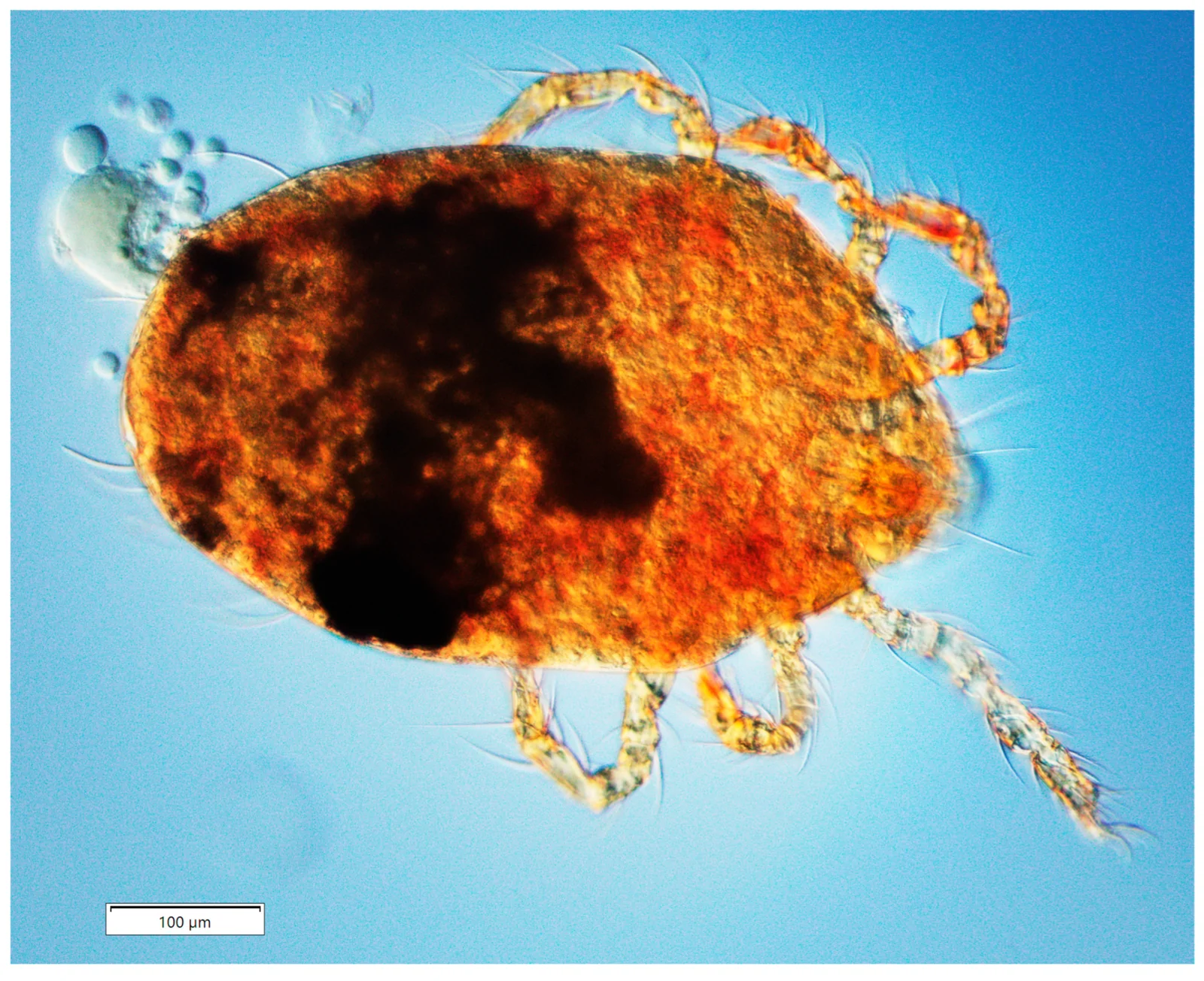
*Blankaartia acuscutellaris* collected from a Bearded Reedling (Mekszikópuszta, Hungary, 2022).

**Figure 5:**
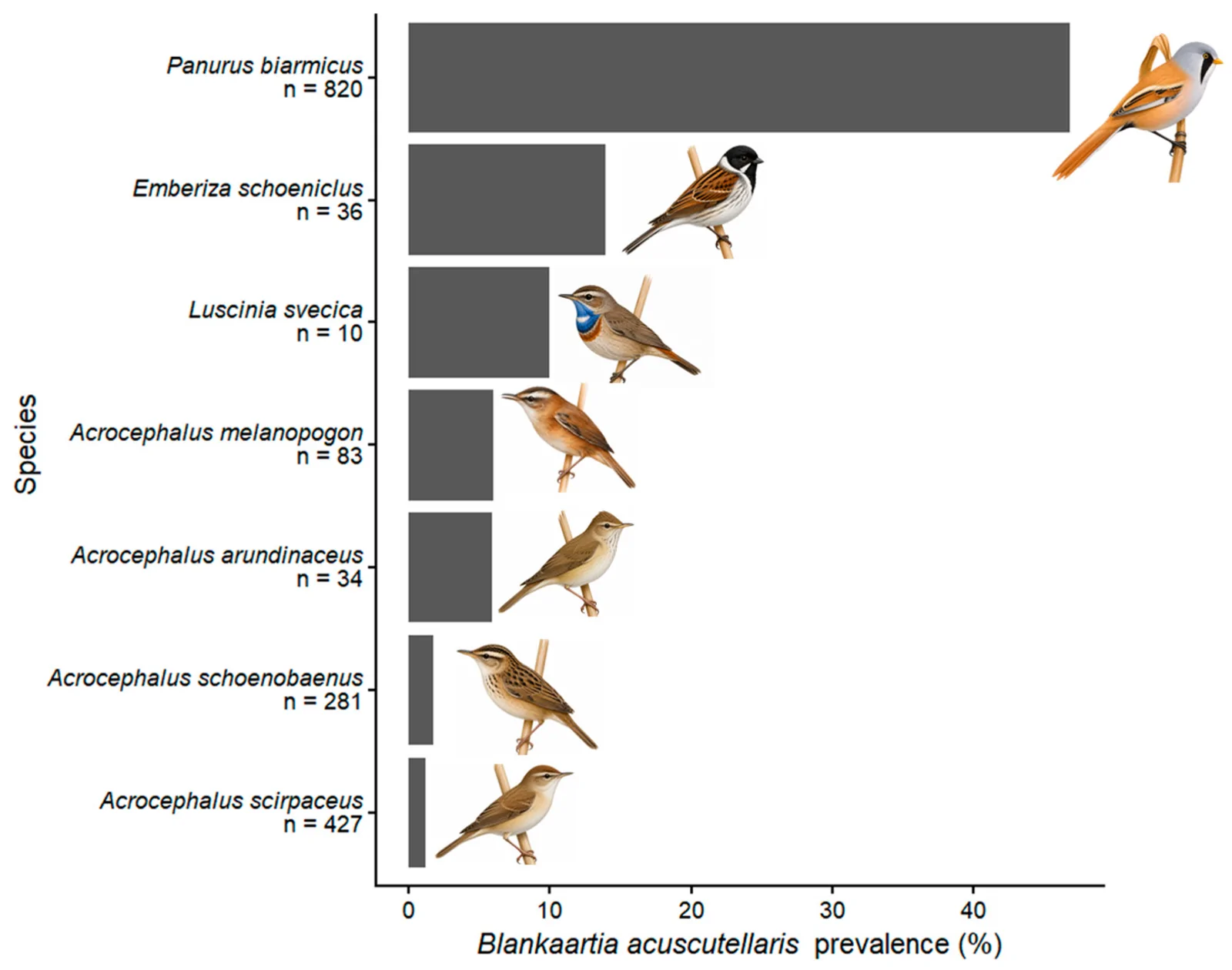
Prevalence of *Blankaartia acuscutellaris* among susceptible bird species at Mekszikópuszta, 2022–2024.

All Trombiculidae specimens collected during the study were identified as *Blankaartia acuscutellaris* (Figure 4), and no other chigger mite species were detected in the examined material. To date, 15 bird species have been recorded as hosts of this mite in Hungary, as summarized previously [26]. The highest prevalence of infestation was recorded in the Bearded Reedling (46.8%), followed by the Common Reed Bunting (*Emberiza schoeniclus*; 13.9%) and the Bluethroat (*Luscinia svecica*; 10.0%). Lower prevalence values were observed in the Moustached Warbler (6.0%), Great Reed Warbler (*Acrocephalus arundinaceus*; 5.9%), Sedge Warbler (1.8%), and Eurasian Reed Warbler (1.2%) (Figure 5).

Infestation rates differed significantly among host species, with Bearded Reedlings showing a significantly higher prevalence (χ^2^ = 451.27, df = 1, *p* < 0.001) compared to all other host species captured at Mekszikópuszta.

In the Bearded Reedling, age classes could be reliably distinguished based on plumage characteristics until late summer [20], which enabled the comparison of *B. acuscutellaris* prevalence between juveniles and adults. During the study period at Mekszikópuszta, age could be determined for 656 individuals. From these, 593 were first-year birds, and 63 were adults. Prevalence of *B. acuscutellaris* infestation was markedly higher in juveniles (361/593; 60.9%) than adults (11/63; 17.5%). The difference in infestation rates between age groups was also statistically significant (χ^2^ = 43.73, df = 1, *p* < 0.001), indicating that juvenile birds are more frequently infected than adult conspecifics.

### 3.2. Phylogenetic Analysis of Blankaartia acuscutellaris

COX1 sequences obtained from three mite samples (HB26, KB70, and HD18) originating from different individuals sampled at different dates were identical at the nucleotide level, and they were combined into a single 582 bp representative sequence for further analysis and submitted to GenBank under the accession number PZ567563.

**Figure 6:**
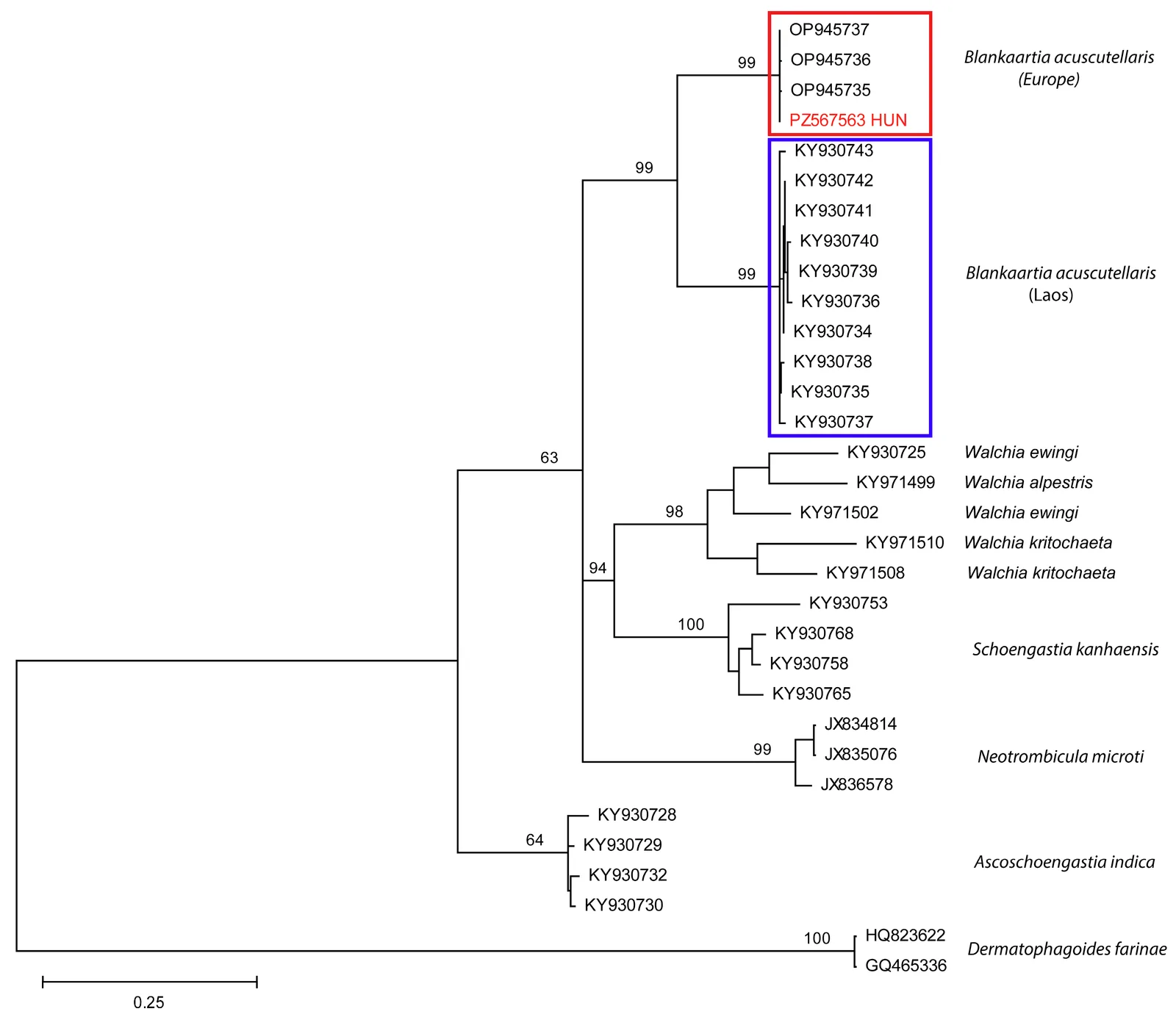
Bayesian phylogeny inferred from 32 arthropod COX1 sequences (582 bp), including Hungarian and Slovakian (red frame) COX1 sequences, and Laotian (blue frame) *B. acuscutellaris* COX1 sequences. The analysis was implemented in MrBayes using the GTR+G+I model of nucleotide substitution. MCMC chains were run for 10,000,000 generations, sampling every 1000 generations, with the first 10% discarded as burn-in. Bayesian posterior probabilities are displayed above the branches, with values ≥ 0.95 indicating strong statistical branch support. The scale bar represents 0.25 substitutions per site.

**Figure 7:**
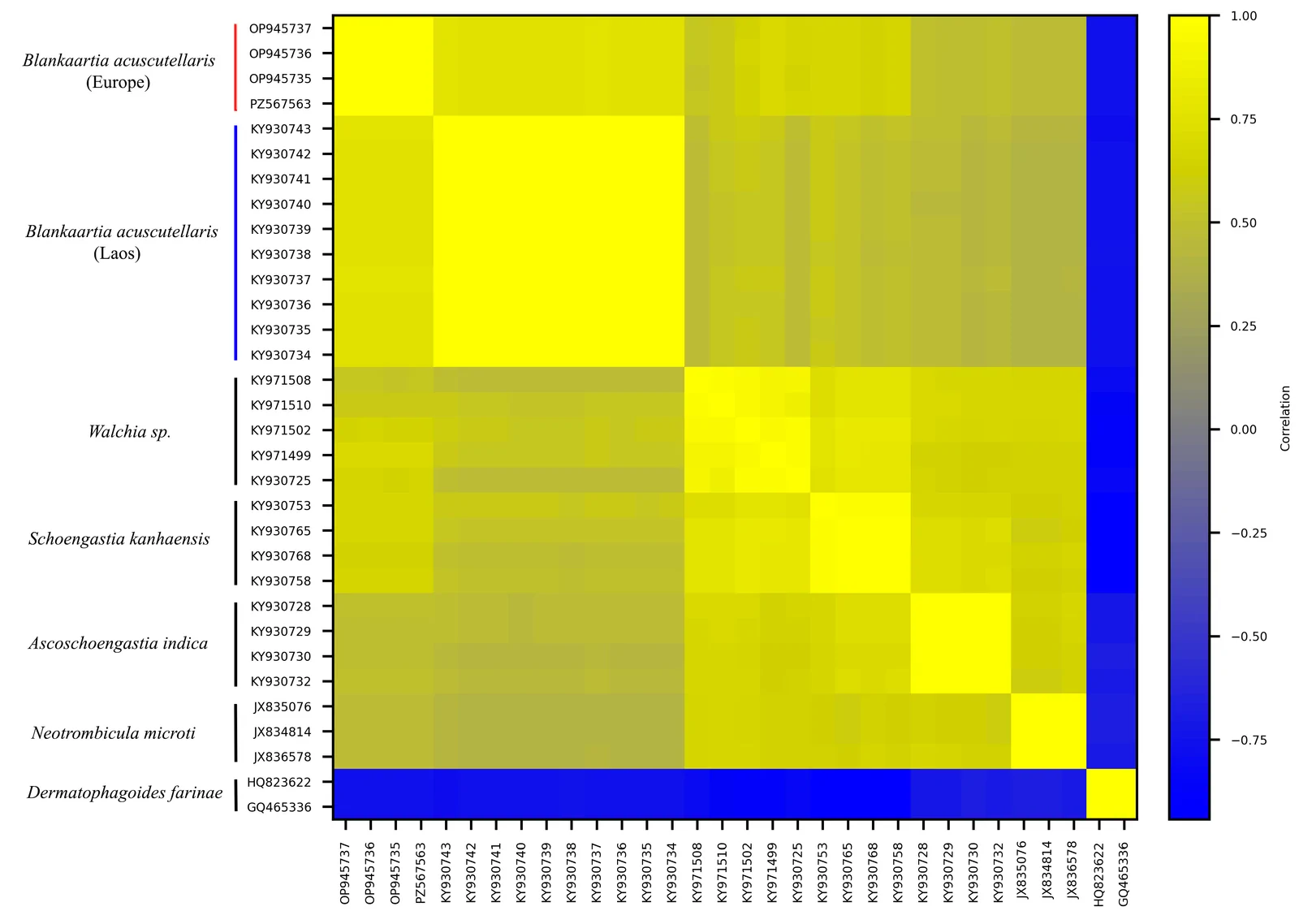
Heatmap of the Spearman Correlation values of pairwise distances obtained from the alignment of Hungarian, Slovakian, Laotian *B. acuscutellaris* and related acariform COX1 sequences visualized in Heatmapper2 application (https://heatmapper2.ca/).

This COX1 sequence was compared to 13 other *B. acuscutellaris* COX1 sequences deposited in GenBank. The Bayesian phylogenetic analysis revealed that our samples formed a well-supported cluster together with samples from Slovakia [18], sharing nearly 100% nucleotide identity. In contrast, sequences from Laos [24] formed a distinct cluster separate from the European *B. acuscutellaris* samples (Figure 6). Pairwise distance analysis and visualization also indicated clear genetic divergence between these groups, reinforcing the phylogenetic separation of *Blankaartia* samples with distinct geographic origins (Figure 7).

Although we established significant genetic divergence based on the COX1 sequence, our samples shared key morphological diagnostic characters of *B. acuscutellaris* with Asian specimens when compared to previously published morphological descriptions and high-quality images.

These results suggest a clear genetic differentiation between Asian and European populations of *B. acuscutellaris* into geographically structured lineages supported by high Bayesian posterior probability values.

In order to provide additional data for compound phylogenetic and taxonomic analyses we have also amplified and sequenced the 18S rRNA region of *B. acuscutellaris* and submitted it to GenBank under the accession number PZ567550. We did not use this ribosomal sequence for direct phylogenetic analysis due to the absence of homologous sequences from conspecific samples.

## 4. Discussion

### 4.1. The Geographical Distribution and Host Spectrum of Harpirhynchus dusbabeki

Earlier reports of *H. dusbabeki* from several European countries, including the Czech Republic, Austria, Croatia, France, Poland, and Slovakia [6], have been complemented by our current findings on its wide distribution in Hungary. Beyond the novel faunistic data, our study provides further insights into the pathogenesis and life-cycle characteristics of *H. dusbabeki* by identifying the presence of its multiple developmental stages within the cutaneous cysts, including adult mites and larvae.

Prior to this study, *H. dusbabeki* had been reported exclusively from a single host species, the Bearded Reedling [4]. Our study demonstrates that, based on current knowledge, at least four bird species in Hungary are susceptible to infestation by *H. dusbabeki,* indicating a broader host spectrum than previously assumed. Our observation aligns with the hypothesis proposed by Moss (1979), who distinguished between cyst-forming and non-cyst-forming species within the genus *Harpirhynchus* [27]. According to this framework, non-cyst-forming species—considered evolutionarily more ancestral—tend to exhibit higher host specificity, often parasitizing a single host species or a narrow host complex. In contrast, cyst-forming species, such as *H. dusbabeki*, are thought to have evolved later and to exploit a broader host range. The evolution of cyst formation may have enabled these mites to utilize multiple host species, reducing host specificity while increasing transmission success and overall fitness [27]. Our results provide empirical support for this hypothesis, demonstrating that *H. dusbabeki* is not restricted to a single host species. Additional host associations will likely be revealed with increased sampling effort.

Despite the apparent ecological and behavioural differences exhibited by identified host species during the breeding season, they all tend to form social groups outside their breeding season [28]. Such aggregations are likely to enhance parasite transmission [29]. Unfortunately, due to the low number of observed cases and the parasite’s sporadic occurrence, we could not assess the seasonal dynamics of *H. dusbabeki* infestation, highlighting the need for further investigation. Previous studies on a closely related species, *H. nidulans*, using bird-ringing data from across Central Europe [2] provide a useful comparative framework. Given the strong morphological similarity and overlapping host spectrum of the two species, it is reasonable to assume that their life cycles are also comparable. Previous observations showed that infestations are absent in juvenile birds [2]. This pattern was reported in multiple countries and confirmed by Literák et al. (2005) in the Czech Republic and Slovakia [3]. Our findings are consistent with these observations, as all detected cases of *H. dusbabeki* infestation occurred exclusively in fully grown individuals. However, the prevalence among juveniles may have been underestimated, as early-stage lesions may not be externally visible, since cyst formation is suggested to be prolonged and likely takes several months. Earlier studies reported a higher prevalence in spring than in autumn, suggesting that lesions may initially be small and difficult to detect during the early stages of development [2]. Larval growth and moulting likely occur in late spring, followed by cyst enlargement in May and June. Peak parasite activity may occur in mid-summer, when cysts become enlarged and may rupture, potentially facilitating transmission between hosts through direct physical contact [2].

### 4.2. The Prevalence of Blankaartia acuscutellaris Among Host Species and Age Groups of Bearded Reedlings

Based on our current knowledge, *B. acuscutellaris* parasitizes at least 15 bird species in Hungary. All affected host species are associated with wetland habitats, particularly reed fields [28], suggesting strong ecological overlap between host populations and the parasite’s preferred microhabitat. Such niche overlap is a key determinant in host–parasite systems [30], as trombiculid mites typically inhabit humid, near-ground environments in marshy areas [9].

Our findings are consistent with previous studies, finding the Bearded Reedling to be the most affected bird, with *B. acuscutellaris* prevalence values of 37.1% in Slovakia [18] and 64.57% in Serbia [31]. Our data collected at Mekszikópuszta showed a similarly high prevalence, finding 46.8% of examined individuals infested, which is likely due to the species’ habitat use and social behavior [18]. This bird species frequently forms aggregations throughout the year, which can enhance parasite transmission through increased contact rates among individuals [32].

Another important result of our study is the pronounced age-related difference in *B. acuscutellaris* infestation rates. Juvenile birds were found significantly more frequently infected than adults, based on longitudinal study design and large sample size. This pattern is consistent with findings reported by Trnka et al. (2023) [18], but contrasts with earlier observations by Márton (1998) [31]. Fledged juvenile Bearded Reedlings form cohesive foraging groups and spend considerable time near the ground in wet reedbeds, where they actively search for food [32]. These habitats coincide with the preferred microhabitat of trombiculid larvae, increasing the likelihood of infestation [9,18]. In contrast, adult individuals may spend less time in such high-risk microhabitats due to ongoing breeding activities, including potential second broods [18,32]. Our results, therefore, lend further support to the hypothesis that differences in behavior and habitat use between age classes play a key role in shaping infestation patterns. The high infection rate observed in juveniles likely reflects increased exposure to parasite-rich microhabitats rather than intrinsic susceptibility alone.

Although the general life cycle of trombiculid mites remains incompletely understood, our findings support the assumption that larval infestation occurs primarily during periods of intensive ground-level activity. Further studies, particularly those targeting different life stages of the mite species, are needed to better understand the full life cycle and transmission dynamics of *B. acuscutellaris*.

### 4.3. Phylogenetics of Blankaartia acuscutellaris

Phylogenetic analysis revealed that COX1 sequences obtained from Hungarian *Blankaartia acuscutellaris* samples clustered together with those from Slovakia. In contrast, morphologically similar specimens from Laos formed a clearly distinct and well-supported clade in the Bayesian phylogenetic tree.

This pattern indicates substantial genetic divergence between geographically distant populations despite their morphological similarity [33]. Pairwise nucleotide distance analysis further corroborated this separation, reinforcing the genetic distinctiveness of these lineages.

Parasitic arthropods are subject to strong selective pressures associated with host exploitation, which may drive rapid molecular evolution while morphological traits remain conserved [34]. This phenomenon has been widely documented in mites, where considerable genetic divergence can occur in the absence of pronounced morphological differentiation [35].

Although further investigation is required, our results suggest that these geographically isolated populations may have followed distinct evolutionary trajectories. Such divergence is consistent with patterns expected under allopatric differentiation, where restricted gene flow between spatially separated populations promotes genetic structuring over time [36].

While we refrain from drawing taxonomic conclusions from the current dataset, the observed discordance between morphological similarity and genetic divergence highlights the potential presence of cryptic diversity in *Blankaartia acuscutellaris*. Further taxonomic advances could be achieved through broader comparative phylogenetic analyses incorporating the *Blankaartia acuscutellaris* 18S sequence published in this study.

This study has several limitations that should be considered when interpreting the results. First of all, molecular analyses could only be successfully completed for *B. acuscutellaris*, as suitable sequence data for *H. dusbabeki* could not be obtained despite several repeated attempts. Secondly, although our dataset provides new insights into bird–mite associations, broader geographic sampling would allow a more comprehensive assessment of the ecological factors influencing mite occurrence, especially for *H. dusbabeki*. Future studies incorporating additional molecular markers and more extensive sampling across host species and regions will further improve our understanding of the ecology, diversity, and evolutionary relationships of mites parasitizing birds.

## 5. Conclusions

Our study demonstrates that host–parasite associations in avian skin mites are more complex than previously recognized. We provide empirical support for the hypothesis that the cyst-forming mite *H. dusbabeki* is less host-specific than previously assumed, while *B. acuscutellaris* uses a broad host range with dominant prevalence in Bearded Reedlings. In addition, the pronounced genetic divergence of geographically separated *B. acuscutellaris* populations highlights the species’ potential cryptic diversity. Together, these findings emphasize the value of integrating faunistic, ecological, and molecular approaches to improve our understanding of the ecology and evolution of parasitic skin mites of birds.

## Figures and Tables

**Table 1:** Sample sizes of captured bird hosts with their mite infestation records.

Bird Species	Captured Individuals	*Blankaartia acuscutellaris* Cases	*Blankaartia acuscutellaris* Prevalence (%)	*Harpirhyncus dusbabeki* Cases
*Panurus biarmicus*	820	384	46.8	10
*Acrocephalus scirpaceus*	427	5	1.2	0
*Acrocephalus schoenobaenus*	281	5	1.8	0
*Acrocephalus melanopogon*	83	5	6.0	0
*Cyanistes caeruleus*	54	0	0.0	0
*Locustella luscinioides*	37	0	0.0	0
*Emberiza schoeniclus*	36	5	13.9	0
*Acrocephalus arundinaceus*	34	2	5.9	0
*Aegithalos caudatus*	21	0	0.0	0
*Sylvia atricapilla*	20	0	0.0	0
*Linaria cannabina*	16	0	0.0	0
*Phylloscopus collybita*	15	0	0.0	0
*Parus major*	12	0	0.0	0
*Luscinia svecica*	10	1	10.0	0
*Passer montanus*	9	0	0.0	0
*Erithacus rubecula*	8	0	0.0	0
*Luscinia megarhynchos*	6	0	0.0	0
*Acrocephalus palustris*	5	0	0.0	0
*Prunella modularis*	3	0	0.0	0
*Remiz pendulinus*	3	0	0.0	0
*Troglodytes troglodytes*	3	0	0.0	0
*Alcedo atthis*	2	0	0.0	0
*Ficedula hypoleuca*	2	0	0.0	0
*Jynx torquilla*	2	0	0.0	0
*Saxicola rubicola*	2	0	0.0	0
*Sturnus vulgaris*	2	0	0.0	0
*Sylvia curruca*	2	0	0.0	0
*Phylloscopus trochilus*	1	0	0.0	0
*Regulus regulus*	1	0	0.0	0
*Sylvia communis*	1	0	0.0	0
*Turdus phylomelos*	1	0	0.0	0
Total	1919	407	21.2	10

## Data Availability

The raw data supporting the conclusions of this article will be made available by the authors on request. All derived sequences of *Blankaartia acuscutellaris* were deposited in NCBI GenBank.

## Supplementary Materials

The following supporting information can be downloaded at https://www.mdpi.com/article/10.3390/biology15171495/s1, File S1: PCR Master Mix and Thermoprofile used for amplification of Blankaartia acuscutellaris COX1 and 18S rRNA genes.
