# Supplementary material for: Integrative Faunistic, Ecological, and Molecular Study of Harpirhynchus dusbabeki and Blankaartia acucutellaris Associated with European Passerines
Source: Biology (Basel). 2026 Sep 2;15(17):1495. doi: 10.3390/biology15171495 (PMC13565479; doi:10.3390/biology15171495)
Supplement: Supplementary file 1 [file biology-15-01495-s001.zip › biology-4529399-supplementary.pdf]

**PCR Master Mix and Thermoprofile used for amplification of *Blankaartia acuscutellaris* COX1 and 18S rRNA genes**

Reference: Klimov et al., 2018 (with in-house modification)

Target gene: mitochondrial cytochrome c oxidase subunit I (COX1 or COI)

FIRST ROUND:

Reaction mixture (25 µL):

5.0 µL 5x Colorless GoTaq Flexi Buffer

3.5 µL $MgCl_2$ 3.5 mM final concentration

1.75 µL dNTP 0.7 mM final concentration

1.0 µL forward primer (COX1_16F) 0.4 µM final concentration

1.0 µL reverse primer (COX1_1324R) 0.4 µM final concentration

0.125 GoTaq DNA Polymerase (5u/µL)

2.5 µL template DNA

Nuclease-free water to the final volume

Primers:

| **Name** | **Sequence (5'-3')** |
|---|---|
| 18S_1F_G | TACCTGGTTGATCCTGCCAGTAG |
| 18S_8r_MH | TAATGATCCTTCCGCAGGTTCACCT |

Thermocycler: Biometra TAdvanced Twin 48G thermocycler (Analytik Jena)

Thermocycling conditions:

Initial denaturation: 95 °C for 1 min

35 cycles of:

- Denaturation: 95 °C for 1 min
- Annealing: 50 °C for 15 s
- Extension: 72 °C for 40 s

Final extension: 72 °C for 5 min

Expected amplicon size: ~1800 bp

SECOND ROUND:

Reference: Klimov et al., 2018 (with in-house modification)

Target gene: mitochondrial cytochrome c oxidase subunit I (COX1 or COI)

Reaction mixture (25 µL):

5.0 µL 5x Green GoTaq Flexi Buffer

3.0 µL $MgCl_2$ 3.5 mM final concentration

1.75 µL dNTP 0.7 mM final concentration

1.0 µL forward primer (COX1_25Fshort_T) 0.4 µM final concentration

1.0 µL reverse primer (COX1_128R_T) 0.4 µM final concentration

0.125 GoTaq DNA Polymerase (5u/µL)

2.5 µL template DNA

Nuclease-free water to the final volume

Primers:

| **Name** | **Sequence (5'-3')** |
|---|---|
| COX1_25Fshort_T | TGTAAAACGACGGCCAGTTCHACWAAYCAYAARRAYA |
| COX1_128R_T | CAGGAAACAGCTATGACCCCWVYTARDCCTARRAARTGTTG |

Thermocycler: Biometra TAdvanced Twin 48G thermocycler (Analytik Jena)

Thermocycling conditions:

Initial denaturation: 94 °C for 2 min

20 cycles of:

- Denaturation: 94 °C for 30 s
- Annealing: 49 °C for 35 s
- Extension: 72 °C for 2 min

18 cycles of:

- Denaturation: 94 °C for 30 s
- Annealing: 52 °C for 35 s
- Extension: 72 °C for 2 min

Final extension: 72 °C for 7 min

Expected amplicon size: ~1200 bp

Reference: Klimov et al., 2018 (with in-house modification)

Target gene: 18S rRNA

FIRST ROUND:

Reaction mixture (25 µL):

5.0 µL 5x Colorless GoTaq Flexi Buffer

3.5 µL $MgCl_2$ 3.5 mM final concentration

1.75 µL dNTP 0.7 mM final concentration

1.0 µL forward primer (COX1_16F) 0.4 µM final concentration

1.0 µL reverse primer (COX1_1324R) 0.4 µM final concentration

0.125 GoTaq DNA Polymerase (5u/µL)

2.5 µL template DNA

Nuclease-free water to the final volume

Primers:

| Name | Sequence (5’-3’) |
|---|---|
| 18S_1F_G | TACCTGGTTGATCCTGCCAGTAG |
| 18S_8r_MH | TAATGATCCTTCCGCAGGTTCACCT |

Thermocycler: Biometra TAdvanced Twin 48G thermocycler (Analytik Jena)

Thermocycling conditions:

Initial denaturation: 95 °C for 1 min

10 cycles of:

- Denaturation: 94 °C for 30 s
- Annealing: 40 °C for 1 min
- Extension: 72 °C for 1 min 40 s

25 cycles of:

- Denaturation: 94 °C for 30 s
- Annealing: 48 °C for 35 s

- Extension: 72 °C for 2 min

Final extension: 72 °C for 7 min

Expected amplicon size: ~1800 bp

SECOND ROUND:

Reference: Klimov et al., 2018 (with in-house modification)

Target gene: 18S rRNA

Reaction mixture (25 µL):

5.0 µL 5x Green GoTaq Flexi Buffer

3.0 µL $MgCl_2$ 3.5 mM final concentration

1.75 µL dNTP 0.7 mM final concentration

1.0 µL forward primer (COX1_25Fshort_T) 0.4 µM final concentration

1.0 µL reverse primer (COX1_128R_T) 0.4 µM final concentration

0.125 GoTaq DNA Polymerase (5u/µL)

2.5 µL template DNA

Nuclease-free water to the final volume

Primers:

| **Name** | **Sequence (5'-3')** |
|---|---|
| 18S_1F_sh_T | TGTAAAACGACGGCCAGTCCTGCCAGTAGTCATATGCTTG |
| 18SR_719R_T | CAGGAAACAGCTATGACCAATATACGCTATTGGAGCTGGA |
| 18S_512F_T | TGTAAAACGACGGCCAGTACATCCAAGGAAGGCAGCAG |
| 18S_1587R_T | CAGGAAACAGCTATGACCAACTAAGAACGGCCATGCAC |
| 18S_1321F_T | TGTAAAACGACGGCCAGTCCCTAGTTCTAACCATAAACGATGC |
| 18S_8R_sh_T | CAGGAAACAGCTATGACCGGTTCACCTACGGAAACCTTG |

Thermocycler: Biometra TAdvanced Twin 48G thermocycler (Analytik Jena)

Thermocycling conditions:

Initial denaturation: 94 °C for 2 min

20 cycles of:

- Denaturation: 94 °C for 30 s
- Annealing: 49 °C for 35 s
- Extension: 72 °C for 2 min

18 cycles of:

- Denaturation: 94 °C for 30 s
- Annealing: 52 °C for 35 s
- Extension: 72 °C for 2 min

Final extension: 72 °C for 7 min

Expected amplicon size: ~720 bp; ~1066 bp; ~600 bp
